# Analysis of the application value of the Roy Adaptation Model in Parkinson's disease nursing care: A non-blinded, single-center observational study

**DOI:** 10.1016/j.clinsp.2025.100820

**Published:** 2025-11-25

**Authors:** Yong-Li Xu

**Affiliations:** Department of Neurology, Guangdong 39 Brain Hospital, Guangzhou, Guangdong Province, China

**Keywords:** Nursing care, Parkinson's disease, Quality of life, Roy Adaptation Model, Self-Efficacy

## Abstract

**Background:**

Nursing care for early-stage of Parkinson's Disease (PD) patients can significantly ameliorate clinical symptoms and enhance quality of life. The Roy Adaptation Model (RAM) improves health status of patients. The objective of the study was to analyze the application value of the RAM in nursing care for patients with PD.

**Methods:**

A total of 272 PD patients (meeting the diagnostic criteria outlined in the “Chinese Guidelines for Parkinson's disease Treatment”; with disease stage I–III) admitted to the institute were selected and randomly divided into a control group (*n* = 136; received routine nursing care for 6-months) and an observation group (*n* = 136; managed using the RAM for 6-months) using a random number table. Participating nurses were required the specialized training requirements (bachelor's degree, 5+ years of experience, RAM certification) for observational group only.

**Results:**

After nursing intervention, the observation group showed significantly lower Webster scale scores in all items, as well as lower Parkinson's Disease Quality of Life Questionnaire scores in all items compared to that of the control group (*p* < 0.05 for all comparisons). The General Self-Efficacy Scale scores in all items, along with nursing satisfaction, were significantly higher in the observation group than in the control group (*p* < 0.05 for all comparisons). Aspiration events for thickened liquids were reduced by 20 % in the observation group whereas those were reduced by 5 % in the control group.

**Conclusions:**

RAM shows potential for application in PD nursing but requires multi-center validation and long-term follow-up to confirm widespread feasibility.

## Highlights

• Parkinson’s Disease (PD) substantially impacts patients' quality of life.
• The Roy Adaptation Model (RAM) was developed on Maslow's hierarchy of needs theory.
• In PD, diagnosis and treatment present significant challenges.
• In PD, RAM may have clinical value.
• RAM improves clinical symptoms, quality of life, and self-efficacy in PD patients.

## Introduction

Parkinson's Disease (PD) is a prevalent neurological disorder primarily affecting middle-aged and elderly populations.^1^ The condition arises from cerebral arteriosclerosis and neurodegenerative changes, characterized by varying degrees of muscular rigidity, tremor, paralysis, and motor dysfunction.^2^ Clinically, due to its slow onset and progressive nature of PD^3^^,^^4^ both diagnosis and treatments present significant challenges, substantially impacting patients' quality of life.^5^

Recent studies have demonstrated that implementing scientific and efficient nursing care for early-stage PD patients can significantly ameliorate clinical symptoms and enhance quality of life.^1^ The Roy Adaptation Model (RAM), developed based on Maslow's hierarchy of needs theory, represents an advanced modern nursing paradigm.^6^ This model not only strengthens individual adaptability but also promotes patients' adaptive development,^7^ ultimately improving their health status. The details of the comparative studies on RAM and/or specialized nursing care in different diseases in different settings are presented in Table 1.

**Table 1:** Details of the comparative studies on the Roy Adaptation Model and/or specialized nursing care in different diseases in different settings.

Study	Published year	Patients’ ethnicity	Sample size (N; eyes)	Age	Main disease(s)	Main conclusions
Case report, Du et al.^5^	2024	Chinese	1	78 years	Dyskinesia hyperpyrexia syndrome	Reducing dopaminergic medications with standard medical care is effective in dyskinesia hyperpyrexia syndrome.
Randomized control study, Zhang et al.^7^	2021	Chinese	120	65 to 85 years (average 74.22±7.97 years)	Hypertension	Nursing intervention based on Roy’s adaptation model enhances self-efficacy, self-management, behaviors, and quality of life, adherence, and compliance of elderly hypertensive patients.
Cross-sectional study, Huang et al.^8^	2024	Chinese	203	≥18-year	Parkinson’s disease	Seasonal variation in temperature may affect motor symptoms in patients with Parkinson’s disease.
Prospective study, Zeng et al.^9^	2022	Chinese	80	Average: 64.21±8.74 years	Parkinson’s disease	Triangle’s tiered and graded management care is effective in Parkinson’s disease patients.
Cross-sectional study, Deal et al.^10^	2019	United States of America	29	Mean: 60.8	Parkinson’s disease	Daily activities instruments evaluate everyday life activities of Parkinson’s disease patients.

Variables presented as mean ± standard deviation.

Although RAM has been effectively implemented in clinical nursing practice across China, research specifically examining its application value in PD patients care remains relatively limited. Therefore, the current study aims to comprehensively investigate and report on the application value of the RAM in PD nursing care.

## Materials and methods

### Ethics approval and consent to participate

This study was approved by the ethics committee of the Guangdong 39 Brain Hospital (Ethics approval number: 20,197,374, dated December 15, 2019), and written informed consent was obtained from all patients and their families before commencement of the study. The study follows the law of China and the v2008 Declarations of Helinski.

### Design, setting and period

A non-blinded, single-center observational study comprised 272 PD patients admitted to the Department of Neurology, Guangdong 39 Brain Hospital, Guangzhou, Guangdong Province, China between January 2020 and December 2023.

### Inclusion/Exclusion criteria

#### Inclusion criteria

1) Meeting the diagnostic criteria outlined in the “Chinese Guidelines for PD Treatment (3rd Edition)”.^11^ 2) Clear consciousness with normal communication ability. 3) Complete general medical records.

#### Exclusion criteria

1) Comorbid metabolic diseases such as diabetes, patients with vascular parkinsonism [Hachinski Ischemic Scale > 7] or mild cognitive impairment [MoCA < 26]) were excluded. 2) Presence of malignant tumors. 3) Severe defects in vital organs, including the heart and brain (e.g., NYHA Class III/IV heart failure, Child-Pugh Class C liver dysfunction) were excluded. 4) Non-compliance with nursing care interventions were excluded.

### Sample size calculation

The study made assumption that the average reduction of 15 % in the Webster scores associated with RAM from previous pilot data and statistical parameters (e.g., *α* = 0.05, *β* = 0.2 for 80 % power). The sample size was 136. G*Power, and Windows software was used to calculate the sample size.

### Groups

**Fig. 1:**
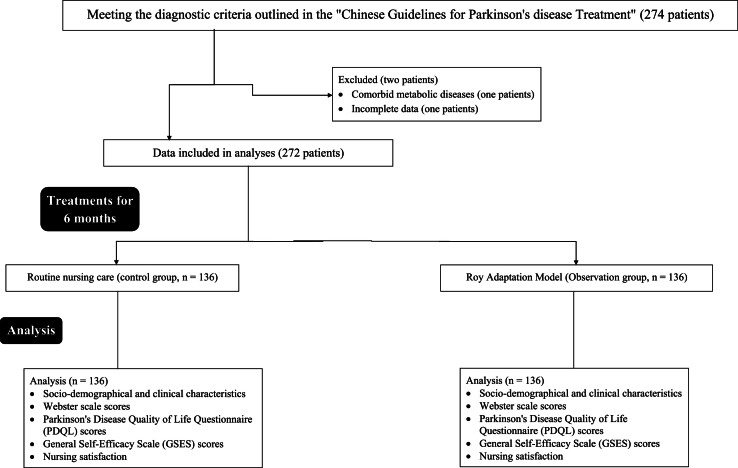
The flow diagram of the study.

Using a random number table method, subjects were divided into a control group and an observation group, with 136 cases in each group. The flow diagram of the study is presented in Fig. 1. Patients were managed on daily care. In a few cases, patients were hospitalized but for fewer days only.

### Interventions

#### Control group

Patients in this group received routine nursing care, which primarily included:

• Health education.
• Disease assessment.
• Clinical indicator monitoring.
• Medication guidance.
• Basic daily care.

Specific interventions included:

• Maintaining appropriate temperature and humidity levels in the ward.
• Real-time monitoring of patients' body temperature and blood pressure.
• Providing routine guidance for emerging issues.
• Assisting patients with proper daily rehabilitation exercises.

The nursing intervention was conducted continuously for 6-months. Five sessions per week, 30-minutes per session, rehabilitation exercises. Specific movements for joint mobility or balance training, like handling object, were performed in rehabilitation exercises. Temperature and blood pressure were measured every week. PD-specific indicators (e.g., UPDRS scores) were monitored by the physician every month. Advice on the timing of levodopa administration relative to meals, management of adverse effects were included in the medication guidance.

#### Observation group

In addition to routine nursing care, this group received the RAM interventions:

1) Level 1 Assessment: Upon admission, nursing staff comprehensively evaluated patients' psychological status through communication and review of pathological records, specifically investigating the presence of anxiety, low self-esteem, and other adverse emotional states (e.g., GAD-7, SDS).^12^

2) Level 2 Assessment: Building upon the Level 1 assessment, this stage involved a detailed analysis of factors contributing to patients' negative emotions. Analysis revealed the following main causes of anxiety and low self-esteem:

• Recognition of extended treatment duration and relatively low cure rates.
• Understanding of high treatment costs and the resulting family financial burden.
• Impact of clinical symptoms on daily life activities and self-care ability.
• Multiple pressures from social and traditional cultural aspects post-diagnosis.

Semi-structured interviews with 136 patients, thematically analyzed by two independent researchers.

3) Nursing Diagnosis: Based on assessment results, patients exhibited varying degrees of low self-esteem, anxiety, and fear, closely related to their family environment, cultural customs, and social context. Nursing interventions focused on addressing:

• Knowledge deficits.
• Nutritional imbalances.
• Decreased self-esteem.
• Physical mobility impairments.
• Family coping and communication barriers.
• Prevention of complications such as pressure ulcers and infections.

4) Establishment of nursing goals

Through RAM implementation, the nursing staff:

• Facilitated effective communication with patients.
• Alleviated negative emotions.
• Enhanced patient adaptation to the treatment environment.
• Established harmonious nurse-patient relationships.
• Improved treatment compliance and confidence in outcomes.

5) Nursing interventions a) Psychological care

• Maintained effective communication with patients.
• Enhanced confidence in treatment and future life.
• Reduced negative emotions related to extended treatment duration.
• Provided clear, professional answers to patient inquiries.
• Establish positive nurse-patient relationships to improve treatment compliance.

b) Safety care

Given the presence of speech and motor function impairments:

• Prevented adverse events such as falls, bed falls, and aspiration (by proper instruction and guidance).
• Enhanced ward rounds
• Provided clear safety instructions to family members
• Encouraged family participation in patient care

c) Dietary care

• Implemented scientific dietary guidance for patients and families
• Conducted comprehensive swallowing assessments
• Provided appropriate dietary recommendations based on individual conditions
• Addressed symptoms such as choking and dysphagia
• The nursing intervention was conducted continuously for 6-months.

### The frequency, duration, and specific content of each RAM intervention

Psychological counseling: 30-minute sessions weekly, using 2–3 PD recovery cases matched to the patient’s age/stage; ward rounds: every 1 h during daytime, every 2 h at night (if patient was hospitalized, otherwise managed on daily care).

### Outcome measures

#### Webster scale assessment

The modified Webster scale was used to evaluate indicators, including self-care ability, gait, and tremor in both groups before and after 6-months of nursing intervention. Higher scores indicate more severe symptoms.^8^

#### Quality of life assessment

The Parkinson's Disease Quality of Life Questionnaire (PDQL) was employed to evaluate quality of life indicators in both groups before and after 6-months of nursing intervention. Higher scores indicate poorer quality of life.^9^

#### Self-efficacy assessment

The General Self-Efficacy Scale (GSES) was utilized to assess indicators, including daily living activities and mental health, in both groups before and after 6-months of nursing intervention. Higher scores indicate better self-efficacy.^10^

#### Nursing satisfaction

After 6-months of nursing intervention, a hospital-designed nursing satisfaction questionnaire was administered to PD patients of both groups. The satisfaction survey used a 100-point scale: 90-points: Satisfied, 60–90 points: Generally satisfied, < 60-points: Dissatisfied.

Total satisfaction rate = Satisfaction rate + General satisfaction rate

(These protocols of hospitals are not published yet). Assessors were blinded to the group.

#### RAM training and quality control

Nurse training consisted of both theoretical and practical components. The theoretical training focused on the specific application of four RAM adaptation modes, while practical training emphasized PD patient assessment methods and intervention techniques. Training requirements included achieving a theoretical examination score of ≥85-points and passing practical operation assessments. The program comprised 24 academic hours of theoretical training and 36 academic hours of practical training, with quarterly updates. Trainers were required to possess RAM certification (for observational group), at least 10-years of neurological nursing experience, and a minimum of 5-years of teaching experience.^13^

Participating nurses were required to hold a bachelor's degree or higher and have at least 5 years of neurological nursing experience. Additional qualifications included PD nursing specialty certification, successful completion of RAM specialized training, and demonstrated competency in communication skills with a strong foundation in psychology.^13^

### Data quality control

Data quality control measures were implemented throughout the study. Data collection was conducted by standardized-trained assessors using standardized assessment tools and procedures, with independent dual-assessor evaluation and inter-rater reliability testing. Data entry utilized a dual-person, dual-computer approach with professional data management software incorporating logic and range verification settings. Quality monitoring included regular sampling of 10 % of data entries, protocols for analyzing and processing abnormal values, and standardized procedures for handling missing data, intention-to-treat analysis.

### Randomization and allocation concealment

An independent researcher was not involved in patient recruitment. The published table was used as the source of the random number table. A patient recruiter held the envelopes for the distribution process of opaque sealed envelopes. The sample size allocation ratio for each Hoehn-Yahr stage was 1:1.

### Statistical analysis

Statistical analysis was performed using SAS 9.4 software. Block randomization was implemented with disease stage (I–III) stratification, using variable block sizes of 4, 6, and 8. Allocation concealment was achieved using opaque sealed envelopes. An independent statistician managed the randomization process, with the sequence stored in a password-protected computer. The Kolmogorov and Smirnov method was used to check the normality of continuous variables. Bartlett's test was used to check the homogeneity of the Standard deviations (SD)s of normally distributed continuous variables. Categorical data were expressed as percentages ( %) and analyzed using Chi-Square tests or Fisher’s test, while measurement data were expressed as mean ± SD and analyzed using *t*-tests. Statistical significance was set at *p* < 0.05.

## Results

### The socio-demographical and clinical characteristics

#### Control group

Seventy males and 66 females; age range 56–78 years, mean age (65.39 ± 4.37 years); disease duration 3–8 years, mean duration (5.92 ± 2.78 years).

#### Observation group

Seventy-two males and 64 females; age range 57–79 years, mean age (66.02±4.48 years); disease duration 3–9 years, mean duration (6.01 ± 2.86 years).

The patients in the control and observation groups have similar UPDRS rating (the UPDRS uses a rating scale from 0 to 4 for most items. Higher scores indicate more severe symptoms. A trained healthcare professional assessed patients' symptoms and assigned scores based on the impairments observed. Medication regimens are comparable for both groups. The demographic characteristics, including disease stage, between the two groups were homogeneous (*p* > 0.05 for comparisons, Table 2), making them comparable for the study.

**Table 2:** Socio-demographical and clinical parameters of the enrolled patients.

Parameters		Cohorts		Comparisons between groups			
		Control group	Observation group				
Non-treatment interventions		Routine nursing care	The Roy Adaptation Model				
Numbers of patients		136	136	*p*-value	Degree of freedom	Test value	95 %CI
Sex	Male	70(51)	72(53)	0.903 (χ^2^-test)	1	0.0147	0.7655 to 1.232
	Female	66(49)	64(47)				
Age (years)		65.39±4.37	66.02±4.48	0.242 (Unpaired *t*-test)	270	1.174	−0.4266 to 1.687
Disease duration (years)		5.92±2.78	6.01±2.86	0.793 (Unpaired *t*-test)	270	0.2632	−0.5833 to 0.7633
Ethnicity	Han Chinese	129(94)	124(91)	0.342 (Fisher-Freeman-Halton test)	N/A	1.384	0.7587 to 2.525
	Mongolian	5(4)	9(7)				
	Tibetan	1(1)	2(1)				
	Uyghur Muslim	1(1)	1(1)				
Education	Primitive	33(24)	32(24)	0.943 (χ^2^-test for Independence)	2	0.1167	N/A
	Below graduate	83(61)	82(61)				
	Graduate or above	20(15)	22(15)				
Hoehn-Yahr Stage	I	69(51)	59(43)	0.257 (χ^2^-test for Independence)	2	2.715	N/A
	II	42(31)	55(40)				
	III	25(18)	22(17)				
Medication regimens	Dopamine agonist	38(28)	35(26)	0.931 (χ^2^-test for Independence)	4	0.8538	N/A
	Dopamine agonist + Sleep problem treatments	55(40)	51(38)				
	Dopamine agonist + gabapentin (Neurontin)	25(18)	30(22)				
	Dopamine agonist + Sleep problem treatments + gabapentin (Neurontin)	15(11)	17(11)				
	Others (not specified by physician)	3(3)	3(3)				

Categorical, continuous normally distributed, and continuous non-normally distributed variables are depicted as frequencies with percentages in parenthesis, mean ± standard deviation, and median (Q3–Q1), respectively.

CI, Confidence interval (using the approximation of Katz for categorical variables), N/A, Not applicable, *χ*^2^, Chi-square.

Test value (χ^2^ value for χ^2^ tests; t-value for *t*-test; relative risk for Fisher test).

### Outcome measures

#### Webster scores

Before the nursing intervention, there were no significant differences in Webster scores between the two groups (*p* > 0.05 for all comparisons). After the intervention, the observation group showed significantly lower scores in self-care, speech, reduced bimanual activity, rigidity, posture, upper limb swing, gait indicators, tremor, facial expression, and rising from the chair compared to that of the control group (*p* < 0.05 for all comparisons; Table 3). Aspiration events for thickened liquids were reduced by 20 % in the observation group whereas those were reduced by 5 % in the control group.

**Table 3:** Comparison of Webster scores between groups.

Items	Time point	Control group, *n* = 136	Observation group, *n* = 136	*t*-value	p-value
Self-care	Before	2.55 ± 0.87	2.56 ± 0.69	0.074	0.941
	After	1.66 ± 0.48	1.43 ± 0.44^a^	2.913	0.004
Speech	Before	2.88 ± 0.82	2.79 ± 0.78	0.656	0.513
	After	1.95 ± 0.55	1.73 ± 0.59^a^	2.249	0.026
Reduced bimanual activity	Before	2.67 ± 0.84	2.62 ± 0.77	0.362	0.718
	After	1.95 ± 0.55	1.48 ± 0.43^a^	5.551	<0.001
Rigidity	Before	2.74 ± 0.67	2.68 ± 0.71	0.507	0.613
	After	1.93 ± 0.56	1.64 ± 0.45^a^	3.329	0.001
Posture	Before	2.71 ± 0.54	2.79 ± 0.46	0.950	0.354
	After	1.85 ± 0.53	1.59 ± 0.52^a^	2.858	0.005
Upper limb Swing	Before	2.77 ± 0.73	2.79 ± 0.69	0.164	0.870
	After	2.48 ± 0.63	1.43 ± 0.42^a^	5.574	<0.001
Gait	Before	2.81 ± 0.69	2.82 ± 0.72	0.683	0.934
	After	2.31 ± 0.55	1.52 ± 0.52^a^	5.468	0.001
Tremor	Before	2.48 ± 0.61	2.52 ± 0.58	0.392	0.969
	After	1.93 ± 0.46	1.76 ± 0.45^a^	2.176	0.031
Facial expression	Before	2.85 ± 0.74	2.79 ± 0.68	0.492	0.623
	After	2.29 ± 0.46	1.71 ± 1.56^a^	2.941	0.004
Rising from the chair	Before	2.87 ± 0.63	2.89 ± 0.67	0.351	0.741
	After	2.55 ± 0.46	1.53 ± 0.48^a^	12.862	<0.001

The table maintains the original format and statistical presentation, with scores shown as mean ± standard deviation. An unpaired *t*-test was used for statistical analyses.

p-values <0.05 indicate statistical significance.

Higher scores indicate more severe symptoms.

The degree of freedom was 270 for all comparisons.

a Significantly lower scores compared that of the control group.

#### PDQL scores

Before treatment, there were no significant differences in PDQL scores between the two groups (*p* > 0.05 for all comparisons). After treatment, the observation group showed significantly lower scores in Parkinsonian symptoms, emotional functioning, and other indicators compared to the control group (*p* < 0.05 for all comparisons; Table 4).

**Table 4:** Comparison of PDQL scores.

Items	Groups	Before Treatment	After Treatment
Parkinsonian symptoms	Control group, *n* = 136	48.95 ± 7.45	37.02 ± 3.55
	Observation group, *n* = 136	49.53 ± 7.19	32.24 ± 0.3^a^
	*t*-value	0.482	15.647
	p-value	0.075	<0.001
Systemic symptoms	Control group, *n* = 136	22.94 ± 4.88	14.12 ± 2.28
	Observation group, *n* = 136	24.24 ± 3.26	13.26 ± 0.69^a^
	*t*-value	1.837	4.21
	p-value	0.070	<0.001
Emotional functioning	Control group, *n* = 136	32.39 ± 5.22	26.23 ± 6.55
	Observation group, *n* = 136	32.63 ± 6.18	24.22 ± 1.23^a^
	*t*-value	0.245	3.517
	p-value	0.807	0.001
Social functioning	Control group, *n* = 136	23.42 ± 6.53	17.63 ± 3.95
	Observation group, *n* = 136	23.85 ± 6.82	15.22 ± 1.43^a^
	*t*-value	0.376	6.69
	p-value	0.708	<0.001

PDQL, Parkinson's Disease Quality of Life Questionnaire.

Lower scores indicate better quality of life.

Values are presented as mean ± standard deviation.

An unpaired *t*-test (two-tailed) was used for statistical analyses.

p-values <0.05 indicate statistical significance.

The degree of freedom was 270 for all comparisons.

a Significantly lower scores compared that of the control group.

#### Self-efficacy assessment

Before treatment, there were no significant differences in GSES between the two groups (*p* > 0.05 for all comparisons). After treatment, the observation group showed significantly higher GSES in daily living activities, compliance behavior, and other indicators compared to the control group (*p* < 0.05 for all comparisons; Table 5). Patients with family caregivers had higher GSES scores than those without (data are not presented in this manuscript).

**Table 5:** Comparison of the general self-efficacy scale.

Items	Groups	Before Treatment	After Treatment	Cronbach’s *α*
Daily living activities	Control group, *n* = 136	75.62 ± 6.94	81.35 ± 7.80	0.86
	Observation group, *n* = 136	75.33 ± 6.89	92.45 ± 8.62^a^	
	*t*-value	0.202	7.846	
	p-value	0.840	<0.001	
Health behavior	Control group, *n* = 136	78.49 ± 6.95	84.52 ± 7.58	0.85
	Observation group, *n* = 136	78.62 ± 7.05	93.15 ± 8.95^a^	
	*t*-value	0.108	6.068	
	p-value	0.914	<0.001	
Compliance behavior	Control group, *n* = 136	70.23 ± 6.79	89.46 ± 7.45	0.83
	Observation group, *n* = 136	69.78 ± 6.75	94.53 ± 7.55^a^	
	*t*-value	0.388	3.803	
	p-value	0.699	<0.001	
Emotional management	Control group, *n* = 136	73.16 ± 7.44	82.31 ± 7.68	0.81
	Observation group, *n* = 136	72.35 ± 7.45	90.82 ± 7.82^a^	
	*t*-value	0.634	6.403	
	p-value	0.527	<0.001	

Higher scores indicate better self-efficacy.

Values are presented as mean ± standard deviation.

An unpaired *t*-test (two-tailed) was used for statistical analyses.

p-values <0.05 indicate statistical significance.

The degree of freedom was 270 for all comparisons.

a Significantly higher scores compared that of the control group.

#### Nursing satisfaction

The nursing satisfaction in the observation group was significantly higher than that in the control group (*p* < 0.05; Table 6). The comments on the results of assumption tests used in the study, with reasons for a conclusive test are presented in Table 7.

**Table 6:** Comparison of nursing satisfaction between groups.

Group	n	Very satisfied	Generally satisfied	Dissatisfied	Total satisfaction rate
Control group	136	60 (44.12)	56 (41.18)	20 (14.71)	116 (85.29)
Observation group	136	80 (58.82)	50 (36.76)	6 (4.41)	130 (95.59)
χ²-value	‒	1.957	0.538	7.037	7.037
p-value	‒	0.190	0.864	0.014	0.014

Total satisfaction rate = ((Very satisfied + Generally satisfied)/Total cases) × 100 %.

The Chi-Square test was used for statistical analyses.

p-values <0.05 indicate statistical significance.

The degree of freedom was one for all comparisons.

χ^2^, Chi-Square.

Variables are presented as frequencies (percentages).

The satisfaction survey used a 100-point scale: 90-points: Satisfied, 60–90 points: Generally satisfied, < 60-points: Dissatisfied.

**Table 7:** Results of assumption tests used in the study, with reasons for a conclusive test.

Parameters	Test
Categorial variables	
2 × 2 Tables	Fisher's exact test or Chi-Square test (if individual sample > 5 and sample size > 40). Chi-Square test with Yates’ corrections if individual sample < 10).
Larger than 2 × 2 cells Tables	Fisher-Freeman-Halton test if fewer than 6 elements in the contingency table per group otherwise Chi-Square of independence.
Continuous variables	
Age (years)	*p* > 0.05 for both columns in the Kolmogorov and Smirnov method and the *p* = 0.387 for Bartlett's test. Therefore, two-tailed unpaired *t*-test was used.
Disease duration (years)	*p* > 0.05 for both columns in the Kolmogorov and Smirnov method and the *p* = 0.371 for Bartlett's test. Therefore, two-tailed unpaired *t*-test was used.
All parameters of Webster scores	*p* > 0.05 for both columns in the Kolmogorov and Smirnov method and the Bartlett's test. Therefore, two-tailed unpaired *t*-test was used.
All parameters of Parkinson's Disease Quality of Life Questionnaire score	*p* > 0.05 for both columns in the Kolmogorov and Smirnov method and the Bartlett's test. Therefore, two-tailed unpaired *t*-test was used.
All parameters of self-efficacy scores score	*p* > 0.05 for both columns in the Kolmogorov and Smirnov method and the Bartlett's test. Therefore, two-tailed unpaired *t*-test was used.

## Discussions

Due to the nature of the disease, PD patients require assistance from others for their daily activities and behaviors, which easily leads to anxiety, depression, self-abasement, and other negative emotions, significantly affecting clinical treatment outcomes.^14^ Some scholars believe that the RAM can notably improve such symptoms. The RAM was first formally proposed by the American scholar Sister Callista Roy,^15^^,^^16^ emphasizing its ability to enhance patient adaptability.

In practical application, nursing staff can identify the causes and specific circumstances of patients' anxiety, self-abasement, and other negative emotions through primary and secondary assessments. Through strengthening patients' cognition and intervention in psychological, physiological, self-concept, and other aspects,^17^ this model can significantly enhance patients' confidence in treatments and prognosis, improving treatment compliance and nursing cooperation.

Special attention must be paid when implementing psychological intervention. After illness onset, patients' daily activities are affected to varying degrees, leading to negative emotions such as pessimism, self-abasement, and anxiety.^18^ Additionally, as the disease progresses and patients learn more about treatments costs and prognosis, their psychological pressure and negative emotions further increase. Therefore, during psychological intervention, nursing staff should:

1. Avoid letting patients focus excessively on the treatments’ negative impacts.
2. Guide patients' attention to the beneficial aspects of treatment by citing successful case examples.
3. Enhance patients' confidence in treatments.
4. Improve their cooperation with nursing care.

Furthermore, based on patients' language and motor function impairment levels, nursing staff should appropriately adjust monitoring frequency and encourage family members to actively participate in daily nursing care,^19^ thereby further improving patients' quality of life during treatments.

In this study, after six months of nursing interventions, the observation group showed significantly lower Webster scores compared to the control group, demonstrating that the RAM effectively improves clinical symptoms in PD patients and has high clinical application value. Additionally, the observation group's PDQL scores were significantly lower than the control group's, indicating that the RAM notably improves patients' motor function and quality of life.

This effectiveness primarily stems from the RAM's ability to improve PD patients' nutritional status through safety management and dietary care, providing fundamental support for clinical treatments. The model also develops targeted exercise intervention programs based on patients' disease stages, effectively preventing and alleviating the severity of physical dysfunction in PD patients.

The intervention strategies vary according to disease stages:

1. Early Stage:
  • Nurses guide patients in appropriate exercises.
  • Ensure adequate joint mobility.
2. Middle Stage (with functional impairment):
  • Planned exercise guidance.
  • Delay progression and worsening of functional disabilities.
3. Late Stage (with significant motor disabilities and long-term bed rest):
  • Passive joint movements.
  • Limb massage.
  • Significantly improve motor dysfunction.
  • Further delay disease progression and deterioration.

Furthermore, the study results showed that the observation group scored significantly higher in self-efficacy indicators, including daily living activities and compliance behavior, suggesting that the RAM effectively enhances PD patients' self-efficacy.^20^ During implementation, nursing staff:

  • Present successful treatment cases.
  • Guide patients to focus on beneficial aspects during treatment.
  • Maintain positive attitudes.
  • Strengthen treatment confidence.
  • Significantly improve treatment and nursing cooperation.

Through increased monitoring and active family participation, patients' self-efficacy was greatly enhanced. The survey results showed that the RAM significantly improved nursing satisfaction among PD patients, reaching 95.59 %. This indicates that the model greatly enhances understanding between nursing staff and patients, facilitating harmonious nurse-patient relationships and improving patient satisfaction with nursing care.

Dietary care in the RAM included swallowing assessments and thickened liquids, reducing aspiration events by 20 % in the patients of the observational group (data are not presented in this manuscript), which may explain lower Webster gait scores.

This study focuses on the application of the RAM in PD nursing, which is clinically relevant. The manuscript exhibits significant flaws in research design rigor, completeness of intervention details, scientific validity of outcome measures, standardization of result presentation, and depth of discussion. However, there are certain limitations to undermine the reliability and reproducibility of the core conclusions. For example, a single-center study with a moderate sample size, the generalizability of results may be limited. Additionally, there is a lack of systematic cost-effectiveness analysis. These limitations suggest future research directions should focus on:

1. Multi-center randomized controlled trials.
2. Development of predictive models.
3. Systematic cost-effectiveness analyses.

To ensure the successful implementation of RAM in clinical practice, healthcare institutions should focus on:

  • Establishing standardized assessment tools.
  • Developing personalized intervention protocol processes.
  • Implementing strict quality control measures.
  • Conducting regular intervention effectiveness evaluations.

In further limitations, the study was conducted at one hospital in China, limiting the generalizability of findings to other populations or healthcare settings (all patients were from one hospital, with 91 %–94 % Han ethnicity, limiting generalizability to diverse populations [e.g., non-Han Chinese, patients in community settings]). While the sample is moderate, its adequacy for detecting clinically meaningful differences is unclear. The 6-month intervention period is insufficient to assess the sustainability of RAM's benefits or its impact on disease progression (PD is progressive, so 6-months cannot assess long-term effects (e.g., whether RAM delays disease progression to Hoehn-Yahr IV/V)). Key variables like medication adherence, comorbidities, or socioeconomic status are not accounted for in the analysis, which could influence outcomes. The specialized training requirements (bachelor's degree, 5+ years of experience, RAM certification) may limit RAM's scalability in resource-limited settings. The study lacks a cost-benefit analysis, which is crucial for healthcare decision-makers evaluating RAM's adoption. The study does not explore subgroup analyses (e.g., by disease severity, age), which could identify patients most likely to benefit from RAM. Outcomes were mostly subjective (PDQL, GSES, satisfaction).

## Conclusions

This study provides strong evidence that the Roy Adaptation Model significantly improves:

  • Clinical symptoms.
  • Quality of life.
  • Self-efficacy in Parkinson's disease patients.

The high satisfaction rates and improved nurse-patient relationships further support its clinical value. The Roy Adaptation Model shows potential for application in Parkinson's disease nursing but requires multi-center validation and long-term follow-up to confirm widespread feasibility. Future research should focus on long-term prognosis and multi-center validation to strengthen these findings.

## Abbreviations

PD, Parkinson's Disease; RAM, The Roy Adaptation Model; PDQL, Parkinson's Disease Quality of Life Questionnaire; GSES, General Self-Efficacy Scale; SD, Standard Deviation; The UPDRS, The Unified Parkinson’s Disease Rating Scale.

## Funding

This research did not receive any specific grant from funding agencies in the public, commercial, or not-for-profit sectors.

## Authors’ contributions

The author has read and approved the manuscript for publication. Yong-Yi Xu was the project administrator and contributed to the conceptualization, the investigation, supervision, resources, methodology, validation, and software, supervision, visualization, and literature review of the study, and contributed to drafting and editing of the manuscript for intellectual content. Yong-Yi Xu agrees to be accountable for all aspects of this work, ensuring its integrity and accuracy. Yong-Yi Xu confirmed the authenticity of the raw data.

## Declaration

All the data and related metadata underlying the reported findings already provided as part of the submitted article. There are no Supplementary Files (supplementary tables, supplementary figures, and others) referred to in the manuscript. Therefore, there are nothing to deposit in appropriate public data repositories.

## Dara availability statements

The data that support the findings of this study are not publicly available because they contain information that could compromise the privacy of research participants, but are available from the corresponding author upon reasonable request.

## Declaration of figures authenticity

All figures submitted have been created by the authors (ore collected from the medical records) who confirm that the images are original with no duplication and have not been previously published in whole or in part.I

## Declaration of competing interest

The authors declare that they have no conflicts of interest or any competing interests regarding the results and/or discussions reported in this research.
